# Renal function after percutaneous cryoablation of renal masses

**DOI:** 10.1590/0100-3984.2020.53.3e1

**Published:** 2020

**Authors:** Sergio Ajzen

**Affiliations:** 1 Full Professor of Radiology, Escola Paulista de Medicina da Universidade Federal de São Paulo (EPM-Unifesp), São Paulo, SP, Brazil. Email: sajzen@terra.com.br.

In the last two decades, there has been a significant increase in the incidental diagnosis of small renal masses by various diagnostic imaging modalities, as well as in the variety of treatments available for such masses. That has led to notable changes in the management of kidney cancer, which has evolved from open surgery to minimally invasive, nephron-sparing procedures. Strategies for the localized treatment of suspicious renal masses include radical nephrectomy, partial nephrectomy, thermal ablation, and active surveillance. The American Urological Association consensus guidelines recently included percutaneous ablation as an acceptable treatment option for high-risk surgical patients^(1)^. The ideal renal tumor for percutaneous thermal ablation is one that measures ≤ 3 cm and is in a location favorable to preserving the largest possible amount of normal renal tissue^(2)^.

In patients diagnosed with a renal mass and submitted to any of the various treatments, the preservation of renal function has become an important consideration. A decrease in renal function is associated with an increased risk of cardiovascular events, hospitalization, and mortality^(3,4)^. It is well established that radical nephrectomy has a significantly greater impact on renal function than does partial nephrectomy^(5,6)^, and that patients undergoing radical nephrectomy are at a higher risk of developing chronic kidney disease^(7)^. However, in studies comparing renal mass ablation and partial nephrectomy, both of which are nephron-sparing approaches, the differences in renal functional changes between the two treatment strategies have not been fully established. Therefore, although ablative techniques can offer advantages such as shorter recovery times and fewer complications, the decline in renal function is still the object of study in relation to the choice of treatments. Given the complexities associated with partial nephrectomy, percutaneous cryoablation can be considered an excellent alternative treatment in selected patients.

In a systematic review and meta-analysis carried out by Patel et al.^(8)^, renal function was assessed after surgical excision, ablation, and active surveillance of localized renal tumors. The authors identified 58 relevant articles and concluded that the implications for renal function varied among management strategies for localized renal masses, postoperative renal function being worse for patients undergoing radical nephrectomy than for those treated with other strategies and the results obtained with thermal ablation being similar to those achieved with partial nephrectomy. They called attention to the need for further studies to quantify changes in renal function associated with active surveillance and nephron-sparing approaches in patients with pre-existing chronic kidney disease.

The current issue of **Radiologia Brasileira** features an interesting article by Staziaki et al.^(9)^ entitled “Early trends and predictors of renal function following computed tomography-guided percutaneous cryoablation of a renal mass in patients with and without prior renal impairment”. In that retrospective study, 39 patients underwent renal mass cryoablation. Despite its limitations (well detailed by the authors), such as the small number of patients evaluated, the retrospective nature of the study, the relatively short follow-up period, and the lack of a control group, their study showed that the trend in renal function after cryoablation did not differ between the patients with previous renal impairment and those without. In addition, the authors found that there were no post-cryoablation changes in the stage of renal disease in either group. They also observed that the glomerular filtration rate at baseline was predictive of the mean glomerular filtration rate in the early post-cryoablation period.

Given the ongoing technological advances in the area of diagnostic imaging and in medical knowledge, we must seek to care for our patients through the use of methods that are increasingly less invasive, in order to obtain the best possible result and with the fewest possible complications. In view of that, we believe that the work conducted by Staziaki et al.^(9)^ makes a noteworthy contribution.

## References

[1] Campbell S, Uzzo RG, Allaf ME (2017). Renal mass and localized renal cancer: AUA guideline. J Urol.

[2] Allen BC, Remer EM (2010). Percutaneous cryoablation of renal tumors: patient selection, technique, and postprocedural imaging. Radiographics.

[3] Go AS, Chertow GM, Fan D (2004). Chronic kidney disease and the risks of death, cardiovascular events, and hospitalization. N Eng J Med.

[4] Tonelli M, Wiebe N, Culleton B (2006). Chronic kidney disease and mortality risk: a systematic review. J Am Soc Nephrol.

[5] Mason R, Kapoor A, Liu Z (2016). The natural history of renal function after surgical management of renal cell carcinoma: results from the Canadian Kidney Cancer Information System. Urol Oncol.

[6] Scosyrev E, Messing EM, Sylvester R (2014). Renal function after nephron-sparing surgery versus radical nephrectomy: results from EORTC randomized trial 30904. Eur Urol.

[7] Kaushik D, Kim SP, Childs MA (2013). Overall survival and development of stage IV chronic kidney disease in patients undergoing partial and radical nephrectomy for benign renal tumors. Eur Urol.

[8] Patel HD, Pierorazio PM, Johnson MH (2017). Renal functional outcomes after surgery, ablation, and active surveillance of localized renal tumors: a systematic review and meta-analysis. Clin J Am Soc Nephrol.

[9] Staziaki PV, Vadvala HV, Furtado VF (2020). Early trends and predictors of renal function following computed tomography-guided percutaneous cryoablation of a renal mass in patients with and without prior renal impairment. Radiol Bras.

